# Address before the Rocky Mountain Med. Ass.

**Published:** 1878-05

**Authors:** 


*Address before the Rocky Mountain Medical Association*, June 6th, 1877, by J. M. TONER, M. D. pp. 122.

It will be remembered that the Rocky Mountain Medical Association consists of those who attended the meeting of the American Medical Association, held at San Francisco, Cal., in May, 1871. The physicians are active, and their friends honorary members.

The address contains observations upon the geological age of the world, the appearance of animal life upon the globe, the antiquity of man, and the archæological remains of extinct races, found on the American continent; also, some very clearly expressed ideas upon the origin and practice of medicine among uncivilized races, more especially the North American Indians.

The address does honor to the author, and is well worth reading by those who interest themselves in the scientific discussions of the age.

